# Orientation Dependent Hardening by <001> Rod-Shaped Misfitting Precipitates in Aluminium Alloys

**DOI:** 10.3390/ma15041380

**Published:** 2022-02-13

**Authors:** Jianbin Liu, Shinji Muraishi

**Affiliations:** Department of Materials Science and Engineering, Tokyo Institute of Technology, 2-12-1 S8-5 Meguro-ku, Tokyo 152-8552, Japan; liujianbin202103@163.com

**Keywords:** aluminium alloys, precipitation strengthening, dislocation dynamics, Eshelby inclusion theory, micromechanics, Green’s function

## Abstract

A coherent precipitate formed in a metallic alloy is of importance in its strengthening mechanism, owing to dislocation/precipitate interaction. Therefore, the present study investigated the effect of <001> rod-shaped precipitates on misfit hardening in aluminium alloys by means of parametric dislocation dynamics simulation based on Green’s function method. The simulation results revealed that the topological evolution of the dislocation microstructure is greatly influenced by local internal stress around the <001> rod precipitate. The strong orientation dependence of misfit hardening was observed for the gradients of the stress–strain curves and their maximum shear stresses, where the difference in the maximum stress values amounted to 30%. The strong and weak hardening behaviours associated with the internal stress of <001> rod precipitates were implemented in terms of the energy associated with the dislocation motion.

## 1. Introduction

Precipitation hardening is an effective approach to improve the mechanical properties of metallic alloys, where the interaction of dislocation with the precipitate results in the higher stress level needed to bypass a precipitate. Basically, it is well known that the precipitation hardening mechanisms are classified into two types: (I) precipitate cut by dislocation and (II) precipitate bypassed to leave Orowan loop, where maximal strength level of the precipitation hardening can be deduced by a combination of these two mechanisms. The above hardening mechanisms and their temperature dependencies by the shearable and non-shearable precipitates were extensively studied in aluminium alloys in the past [1,2], which are extremely important for the prediction of macroscopic elasto-plastic deformation in the engineering process [3].

However, the strengthening effect by misfitting precipitates of certain shapes has not been fully understood in relation to their geometry-dependent hardening. If a precipitate exhibits mismatches in lattice constant, elastic stiffness and plastic strain, internal stress around the precipitate should be taken into account for dislocation interaction with the precipitate. According to the micromechanics theory [4], internal stress due to the different types of mismatches can be formulated in terms of eigenstrain (stress-free strain), which is inhomogeneously distributed in the solid. In the case of the lattice mismatch, the elastic field caused by the misfitting precipitate is not influenced by plastic deformation (Eshelby inclusion problem). In contrast, in the case of the precipitate with dissimilar stiffness, stress disturbance under external and internal stresses is proportional to the elastic strain acting on the precipitate (Eshelby inhomogeneity problem), which can be deduced in the equivalent inclusion method by assuming fictitious eigenstrain. The last case of the mismatch of plastic strain is similar to the first case; however, the internal stress around the precipitate is proportional to the plastic strain [5]. Accordingly, the strengthening effect caused by the lattice misfit is stationary from the beginning of plastic deformation and influences macroscopic yield stress. Meanwhile, the strengthening effect by the plastic strain mismatch influences the linear work hardening of plastic flow. The linear work hardening by non-deformable particles was discussed in terms of the Eshelby inhomogeneity problem by Tanaka and Mori [6] and strengthening associated with the dislocation arrangement around the θ′ plate precipitates in the Al–Cu alloy was reported by Russell and Ashby [2]. Recently, the evidence of elastic distortion in non-shearable precipitates among the plastically deformed matrix phase was experimentally determined in an in situ neutron diffraction study for the lattice strain measurement of a θ′ precipitate and matrix phase under tensile loading in an Al–Cu alloy [7]. Even in the case of shearable precipitates, the formation of an Orowan loop was found in the vicinity of the θ″ platelet (GP-II zone) in the Al–Cu alloy by micropillar compression tests [8]. These pieces of evidence strongly suggest the importance of internal stress in the dislocation motion around the misfitting precipitate. In fact, maximum strengthening levels for age-hardened aluminium alloys were achieved by densely distributed fine misfitting precipitates.

Although many studies associated with precipitation hardening in aluminium alloys have been reported [1,2], it is hard to measure the strengthening effect by a certain precipitation variant, especially for the GP-zones and the fine meta-stable precipitates typically formed in aluminium alloys. It should be noted that orientations and shapes of precipitation variants are correlated with types of misfit strains, because the nucleation and growth of the precipitates take place in order to minimise their strain energy: biaxial misfit strains along transverse directions of <001> rods in Al–Mg–Si alloys [9,10,11], large negative misfit strains normal to {001}GP-zones and θ′ plates in Al–Cu alloys [12,13], {111} Ω plates in Al–Cu–Mg–Ag alloys [14,15], etc. It is interesting to note that under external stress during ageing treatment, the stress orienting effect of precipitation variants occur due to the interaction energy between the external stress and the misfit strain [16,17,18]. By applying the stress ageing method, strengthening by single-variant GP-zones in stress aged Al–Cu single crystals was experimentally determined by Eto [19] and Muraishi et al. [20]. They reported that the yield and flow stresses in stress-oriented GP-zones were remarkably different depending on the dislocation cutting geometry of GP-zone variants. Herein, GP-zones were strengthened when the dislocation Burgers vector was parallel to the GP-zone plates. Following their experimental results, Singh et al. analysed the dislocation interactions of single-variant GP-zones by molecular dynamic simulation, where the strengthening effect by a single-variant GP-zone varied depending on the edge and screw components of dislocation segments [21]. More importantly, they suggested the importance of the cross-slip event without thermal activation at a low temperature (0 K). This demonstrates that the dislocation motion around the misfitting precipitates is greatly influenced by the internal stress caused by the precipitates. Therefore, the internal stress effect on the precipitation hardening should be reconsidered in terms of the orientation and geometry-dependent hardening by precipitation variants. Although the abovementioned research manifested strengthening by the plate-shaped precipitates, the analysis of the misfit hardening effect by rod- and needle-shaped precipitates by experiments and simulations is important.

Owing to the recent development in computational dislocation dynamics [22,23,24], topological evolution and hardening associated with dislocation interactions in bulk and nanostructured materials have been simulated by many researchers [25]. The present study employed parametric dislocation dynamics (PDD) proposed by Ghoniem [26], where the elastic field of arbitrary curved dislocation lines was numerically simulated by the line integral of Green’s functions. In order to determine the dislocation interaction with the internal stress field of precipitates, computation of the stress caused by the misfitting precipitate is mandatory. Takahashi and Ghoniem reported the precipitation hardening of spherical inhomogeneity by means of the PDD method, where the stress disturbance due to the inhomogeneity accounted for the dislocation interaction with the precipitate by the boundary integral equation method [27]. The phase-field dislocation simulation of the Orowan strengthening by a coherent θ′ plate in an Al–Cu alloy revealed that the effect of the coherent strain of non-shearable θ′ plates on the critical resolved shear stress (CRSS) is marginal compared to the simulation models of θ′ plates with and without coherency strains [28]. The discrete dislocation dynamic simulation of the interaction of an edge dislocation with a *θ*′ precipitate in an Al-Cu alloy also found that the elastic mismatch has a negligible influence on the dislocation–precipitate interaction [29]. The precipitation strengthening of β″ needles in Al–Mg–Si alloys, simulated by discrete dislocation dynamics, demonstrated that matrix misfit stresses, volume fraction and precipitate shape have a small effect on the CRSS. Therefore, the Orowan looping only sets an upper bound for CRSS, even at peak ageing condition [30].

Recalling the aforementioned micromechanics theory [4], the stress acting on the dislocation segments can be readily computed by solving the Eshelby inclusion and inhomogeneity problems. Recently, we proposed the micromechanical based Green’s function method for dislocation interaction with the misfitting precipitate [31,32]. The stress of the dislocation segments was numerically computed by the method analogous to the PDD, while the stress inside and outside the precipitate was determined by the Eshelby method. Another important feature of our method is the prediction of the stress–strain curves during the dislocation bypassing the precipitate and the interaction energy analysis [31,32]. Hence, it is crucially important to investigate the orientation-dependent hardening by precipitation variants with different shapes by dislocation dynamics simulation.

The aim of the present study is to investigate the effect of geometry and orientation of the <001> rod precipitate on the misfit hardening behaviour by means of dislocation dynamics simulation. To this end, the hardening and softening behaviours associated with the dislocation motion around the <001> rod precipitates are implemented by the energy associated with the dislocation motion, as proposed in our previous papers [31,32].

## 2. Theory

### 2.1. Geometry of Dislocation Interaction with a <001> Rod Precipitate

**Figure 1:**
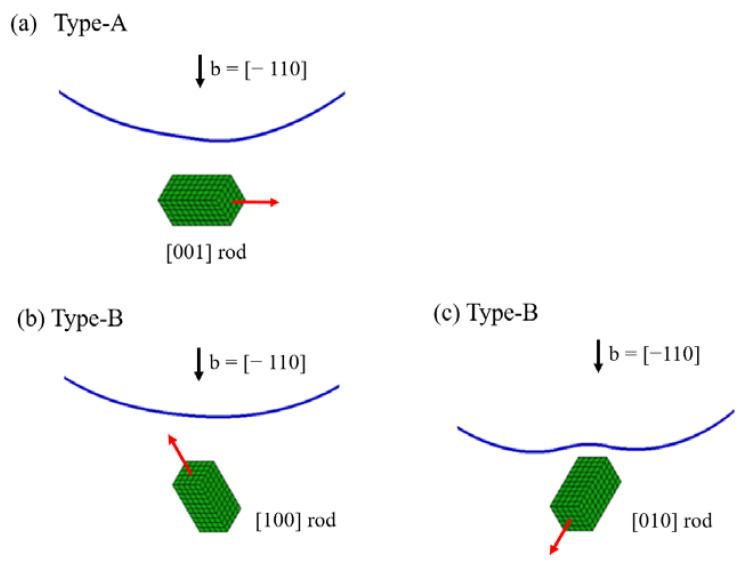
Rods and dislocation on the (111) slip plane. Type-A variant: dislocation Burger’s vector perpendicular to the [001] rod in (**a**). Type-B variants: dislocation Burger’s vector intersecting [100] and [010] rods with an inclined angle in (**b**,**c**), respectively.

There are three different geometries in dislocation interactions with <001> rods, as shown in Figure 1, which are classified into Type-A and Type-B variants as follows. In the Type-A variant, the longitudinal direction of the rod is normal to the dislocation Burgers vector (a). In the Type-B variants (b) and (c), the Burgers vector intersecting a rod is inclined. According to the analysis of interaction energy between dislocation and <001> rods (Appendix A), the average interaction energy becomes zero in the case of the Type-A variant when the biaxial misfit strain is assumed for the rod (misfit strain along the transversal direction of the rod). On the contrary, opposite signs of interaction energies exist in the Type-B variants. The above facts arouse the importance of the orientation dependent hardening effect by <001> rod precipitates, where internal stress due to the <001> variants may cause hardening and softening effects on the macroscopic stress–strain responses under dislocation and precipitate interactions. Note that dislocation interaction with {001} plates is also absent in the Type-A variant, with the misfit strain perpendicular to the plate. Therefore, the general tendency of the dislocation and precipitate interactions can be classified by the dislocation cutting manners of precipitation variants (Type-A and Type-B).

### 2.2. Computation of Dislocation Motion among Internal Stress of a <001> Rod

**Figure 2:**
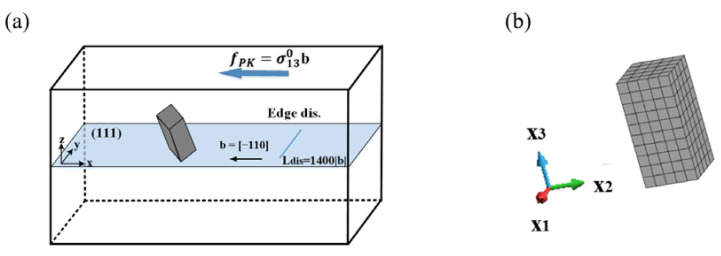
The geometry of dislocation interaction with a [001] rod precipitate. (**a**) <001> rod and edge dislocation in x-y-z global (dislocation) coordinate. (**b**) Discretized [001] rod precipitate in x_1_-x_2_-x_3_ local (precipitate) coordinates.

Figure 2 shows the schematic of the simulation model of an edge dislocation interaction with a [001] rod precipitate. According to the small mismatch of stiffness of aluminium and β″ needle precipitates in Al–Mg–Si alloys [10], the stress disturbances around the inhomogeneity under external and internal stresses are relatively small. Therefore, the internal stress was computed by the Eshelby inclusion problem method, by assuming the matrix stiffness and aluminium as a precipitate phase. The simulation method in the present study is analogous to that in our previous reports [31,32], where the dislocation motion influenced by the stresses of the misfitting precipitate and the curved dislocation segments are explicitly calculated by Green’s function method. The related parameters of the simulation model are listed in Table 1.

As shown in Figure 2a, the primary slip system is selected as (111) slip plane and [−110] slip direction. For ease of plastic strain calculation by dislocation motion in infinite elastic solid, the finite simulation volume of 7.0 × 10^−20^ m^3^ was assumed in the x-y-z coordinates (Δx=2000|b|, Δy=1500|b| and Δz=1000|b|). Crystal orientation of x, y and z directions are along [1 −1 0], [1 1 −2] and [1 1 1], respectively. The <001> variant with the size of a=119|b|, h=238|b| (aspect ratio: α = 2) was placed at the origin of the coordinate. The biaxial eigenstrain was assumed for the rod precipitate, for instance, the misfit strain of the [001] rod in the local coordinate (Figure 2b) is given by $\varepsilon_{11}^{*}=\varepsilon_{22}^{*}=0.05,\varepsilon_{33}^{*}=0$. By applying the rotation matrix $A^{R}$ (Appendix A), the stress of the certain <001> rod in the local coordinate, $\sigma^{\prime }$, was transformed into that in the global coordinate on the dislocation slip plane, σ. Note that in view of the external work carried out (potential energy), the <001> rod interacts with the applied stress differently depending on the rod orientation. This indicates that the magnitude of the external work conducted for each of the <001> rods is varied with respect to the direction of plastic deformation. The details of the average interaction energy for {001} rods are also listed in the Appendix A.

Due to the present geometry of dislocation and precipitate, the influence of the internal stress field on the dislocation bowing out can be analysed. By setting the direction of the straight edge dislocation parallel to the *y*-axis at x=600|b|, where both ends of the dislocation segments are fixed, the dislocation source with the length of 1400|b| was assumed. The dislocation motion behaviours, stress–strain curves and interaction energies were simulated under the condition of remotely applied stress, $\sigma_{13}^{0}$, and a constant strain rate of $\dot{\varepsilon }=\varepsilon /\Delta t=1000\;s^{-1}$. The stress and energy formulations in terms of Green’s functions can be found in our previous papers [31,32].

## 3. Results

### 3.1. Stress Field of <001> Rod Precipitate on the (111) Dislocation Slip Plane

**Figure 3:**
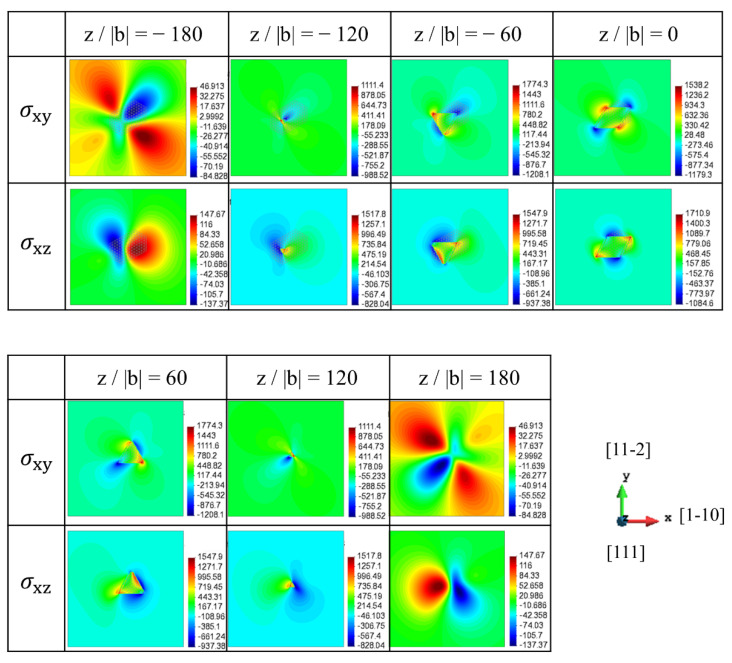
Stress field of [100] rod on the (111) slip plane (Type-B variant). $\sigma_{\mathrm{xy}}$ and $\sigma_{\mathrm{xz}}$ in dislocation coordinate are represented at the different positions (z/|b| = −180 to 180).

**Figure 4:**
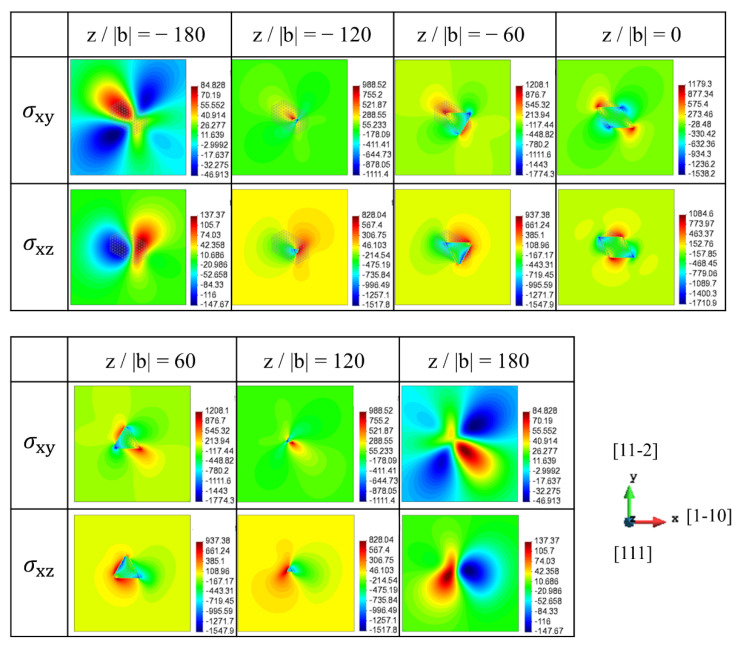
Stress field of [010] rod on the (111) slip plane (Type-B variant). $\sigma_{\mathrm{xy}}$ and $\sigma_{\mathrm{xz}}$ in dislocation coordinate are represented at the different positions (z/|b| = −180 to 180).

**Figure 5:**
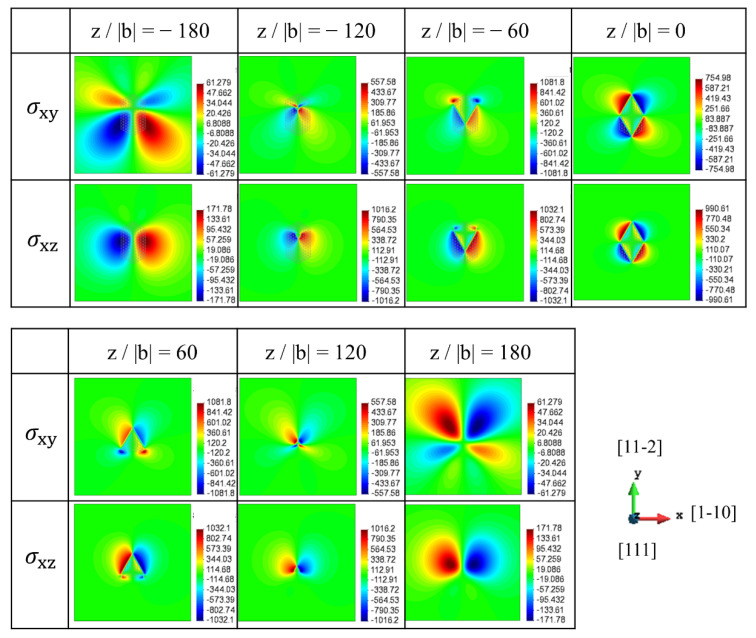
Stress field of [001] rod on the (111) slip plane (Type-A variant). $\sigma_{\mathrm{xy}}$ and $\sigma_{\mathrm{xz}}$ in dislocation coordinate are represented at the different positions (z/|b| = −180 to 180).

The stress fields of the [100] rod precipitate on the (111) slip planes are represented in Figure 3, where several x-y cross-sections of the rod precipitate are chosen among z/|b| = −180 to 180. The projection length of the [100] rod precipitate ranged from z/|b| = −137 to 137. In the present dislocation slip geometry, $\sigma_{\mathrm{xz}}$ and $\sigma_{\mathrm{xy}}$ are associated with dislocation glide and cross-slip, respectively. Note that the magnitudes of contour plots in Figure 3, Figure 4 and Figure 5 are displayed in units of MPa, where the minimum and maximum stress values measured on each of the slip planes are presented by a colour-coded bar. Apparently, the stress magnitude in Figure 3 within the cross-section of the rod precipitate (−120 < z/|b| < 120) is larger than that outside the rod. More importantly, the signs of $\sigma_{\mathrm{xz}}$ and $\sigma_{\mathrm{xy}}$ are varied with the relative height of the slip plane, z/|b|. The large magnitude of $\sigma_{\mathrm{xz}}$ with positive and negative signs can be seen to the right and to the left of precipitates at z/|b| = −120 and −60, respectively, while the opposite picture is observed at z/|b| = 60 and 120. Therefore, the stress distribution around the precipitate has two-fold symmetry with respect to the *z*-axis. This indicates that the sign of dislocations interacting with the [100] rod is turned opposite across the mid-plane, z/|b| = 0. Note that in the case of spherical precipitate with dilatational eigenstrain, the stress on the mid-plane becomes zero [31]. On the contrary, the magnitude of the stress associated with the cross-slip, $\sigma_{\mathrm{xy}}$, is comparable to $\sigma_{\mathrm{xz}}$. Considering that the cross-slip direction is lying in the secondary slip plane, the cross-slip is possible by the screw segment when the force along the secondary slip is larger than that along the primary slip.

Figure 4 shows the stress fields of the [010] rod precipitate on the (111) slip plane, where the stresses in the dislocation coordinate are displayed at the position ranging from z/|b| = −180 to 180. Compared to the internal stresses of the [100] precipitate (Figure 3), the stress spatial distributions in the slip plane show mirror symmetry with respect to the x plane and the sign of the stress becomes opposite due to the geometrical relation of the [100] and [010] rods. Therefore, although the dislocation cutting geometries of the [100] and [010] rods are classified as the Type-B variant (Figure 1), the moving dislocation is differently affected by the local negative and positive interactions. This fact is confirmed by the average interaction energy between the eigenstrain, and the stress associated with dislocation glide, as represented in Appendix A, where the interaction energy becomes opposite signs for two Type-B variants.

The stress fields of the [001] rod precipitate on the (111) slip planes are represented in Figure 5, where the stresses in the dislocation coordinate are displayed at the position ranging from z/|b| = −180 to 180. Note that the spatial distribution of the stresses is totally different from those for the [100] and [010] rod precipitates because the dislocation Burger’s vector [−110] and the [001] rod precipitate are orthogonal. Therefore, $\sigma_{\mathrm{xz}}$ of the same magnitude but the opposite sign is evenly distributed around the [001] rod precipitate, especially on the mid-plane, z/|b| = 0. As represented in Appendix A, the interaction energy of the dislocation and the [001] rod precipitate disappears because of the symmetric distribution of opposite signs of the stresses on the slip plane, which is a characteristic feature of the Type-A variant. Although the details are not mentioned in this paper, compared with the stress field of the {111} plate-shaped precipitate [32], the $\sigma_{\mathrm{xz}}$ magnitude of the {001} rod precipitate is larger than that of the {111} plate precipitate when the total sum of eigenstrain is assumed as a constant value, $\varepsilon_{11}^{*}+\varepsilon_{22}^{*}+\varepsilon_{33}^{*}=0.1$. However, the average interaction energy for the rod precipitate is smaller than that for the plate-shaped precipitate, which is $E_{\left[100\right]}^{int}=0.0204\;\sigma_{\mathrm{xz}}$ for rod and $E_{\left(-111\right)}^{int}=0.0272\;\sigma_{\mathrm{xz}}$ for plate, respectively. This implies that the strong positive and negative stress $\sigma_{\mathrm{xz}}$ is localised around the rod precipitate. Another important fact addressed here is that the $\sigma_{\mathrm{xy}}$ stress field for cross-slip is relatively smaller than $\sigma_{\mathrm{xz}}$ when the rod-shaped precipitate is considered. This indicates that the glide motion is preferable to the cross-slip under dislocation interaction with the rod precipitate.

### 3.2. Dislocation Motion Behaviours around <001> Rod Precipitates

**Figure 6:**
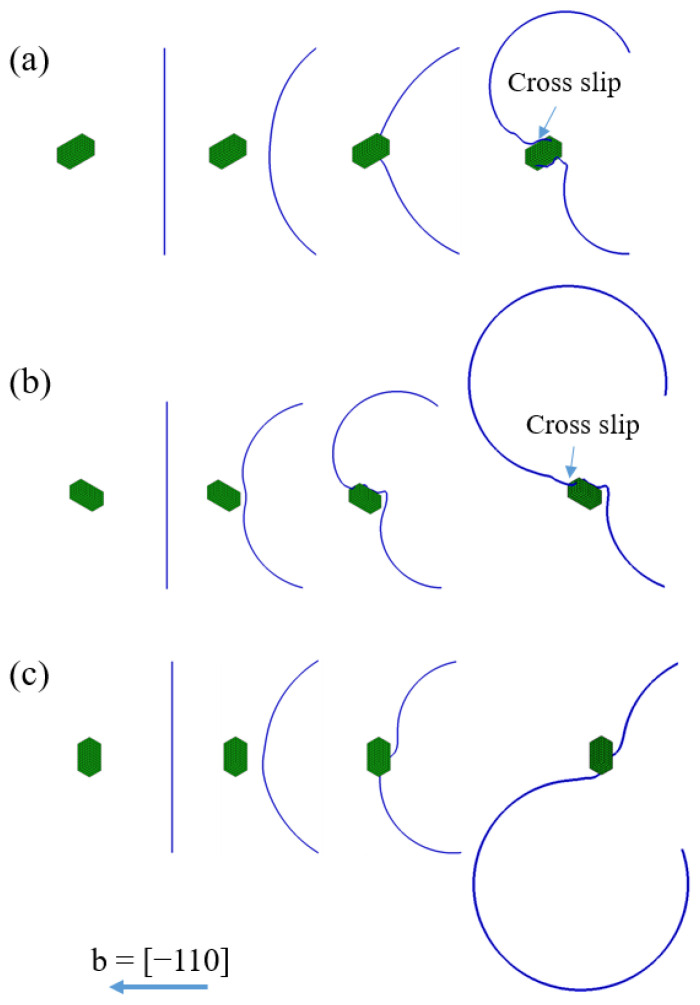
An edge dislocation bypassing a rod precipitate with orientation of (**a**) [100], (**b**) [010], (**c**) [001]. Dislocation lies in the slip plane at z = 0.

Figure 6 shows the dislocation motion behaviours around the <001> rod precipitate with different orientations, where the edge dislocation bypasses each of the <001> rod precipitates. The dislocation slip plane is assumed at z/|b| = 0. Apparently, it can be seen that the dislocation interacts with the <001> rod precipitates in a different way, depending on the rod precipitate orientation. Regarding the [100] rod precipitate in Figure 6a, the dislocation cuts into the precipitate because the stress field of $\sigma_{\mathrm{xz}}$ inside the [100] rod precipitate is positive, as shown in Figure 3. However, the dislocation motion is retarded since the stress outside the precipitate is negative on the slip plane (z/|b| = 0, upper and lower sides of the rod precipitate in Figure 3). As the external stress is increased, the upper side of the dislocation arm overcomes the precipitate to change the movement direction.

Meanwhile, the dislocation motion around the [010] rod precipitate in Figure 6b is different from the previous example, because the respective stress fields of $\sigma_{\mathrm{xz}}$ inside and outside the precipitate change their signs (Figure 4). Therefore, the dislocation cannot cut into the precipitate and further dislocation motion would occur at the positive stress field around the precipitate (Figure 6b).

For the precipitate with [001] orientation, the positive and negative stress field of $\sigma_{13}$ coexist in front of the precipitate (Figure 4), so the lower side of the dislocation arm is attracted by the strong positive stress field, but the upper side of the dislocation arm is repelled by the negative stress field, as shown in Figure 6c. Note that, as represented in the Appendix A, assessment of the average interaction between external stress and eigenstrain in Equation (A4) suggests that the hardening and softening behaviours exist in the dislocation interaction with the [100] and [010] rod precipitates, while the interaction energy is absent in the dislocation interaction with the [001] rod precipitate. In view of the dislocation topology, the swept area of the dislocation seems smaller in Figure 6a under the similar simulation time step. This indicates that the resultant dislocation velocity is decreased by the misfitting precipitate (Figure 6a); hence, the external stress level should be increased to maintain the constant strain rate condition. Therefore, the interaction force acting on the dislocation effectively retards the bypassing process.

### 3.3. Orientation Dependent Hardening by <001> Rod Precipitates

**Figure 7:**
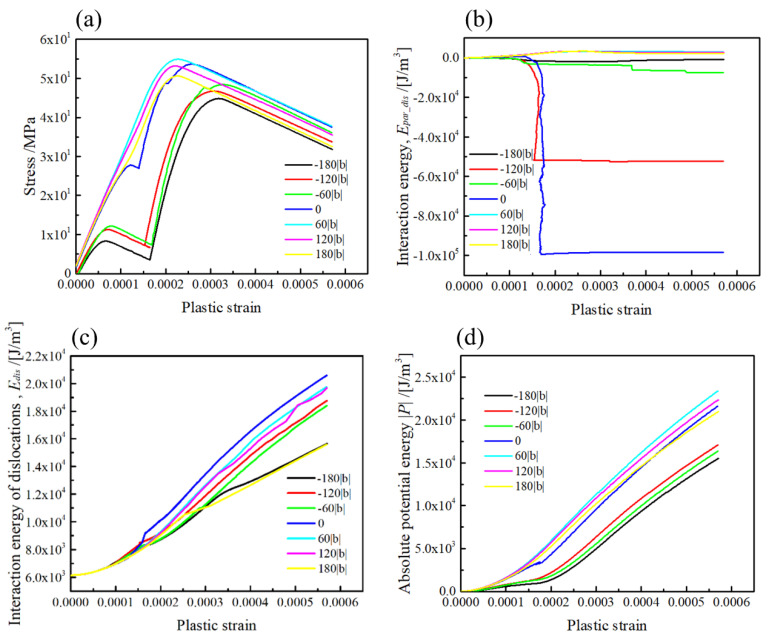
The geometrical effect on the misfit hardening by the [100] rod precipitate. (**a**) Stress–strain curve, (**b**) interaction energy of the precipitate and dislocation, (**c**) interaction energy of the dislocations and (**d**) potential energy due to the dislocation motion The relative position of the (111) slip plane is ranging from z/|b| = −180 to 180.

The computation results on the stress–strain curves, and energies of the dislocation interaction with the [100] rod precipitate, are represented in Figure 7 (Type-B variant). The plastic strain is computed from the area swept by the dislocation [4]. Note that the interaction energy of dislocation is the sum of its own energy and interaction energy of dislocation segments. It is obvious that the stress required for dislocation bypassing the rod precipitate largely depends on the relative position of the slip plane (Figure 7a), where the initial hardening linearly increased for the slip plane positions at z/|b| > 0. Meanwhile, the stress remarkably drops on the stress–strain curves for the slip planes at z/|b| ≤ 0.

The effects of misfit hardening and dislocation strengthening on the stress–strain curves can be discussed in terms of the interaction energy between the dislocation and precipitate (Figure 7b), and between the dislocation and dislocation (Figure 7c), respectively. When the slip plane is above the mid-plane of the precipitate at z/|b| > 0, the interaction energy of the dislocation and precipitate is positive throughout the profile, with a maximum value of up to 3 × 10^3^ J/m^3^. Meanwhile, the interaction energy suddenly drops for the slip plane at z/|b| = 0 and below z < 0. Since the hardening caused by the dislocation–precipitate interaction is attributed to the negative sign of $\sigma_{\mathrm{xz}}$, the dislocation motion is always retarded in front of the precipitate for the slip plane at z/|b| > 0. Therefore, the decrease in the interaction energy indicates that the dislocation motion on the slip plane at z/|b| < 0 is promoted by the positive stress in front of the precipitate. Hence, the flow stress strongly drops in the initial part of the stress–strain curves. Regarding the interaction energy of dislocations in Figure 7c, the largest one is achieved in the slip plane at z/|b| = 0, which reflects the increase in the dislocation interaction energy at the dislocation topological change, as shown in Figure 7a. Accounting for the fact that the dislocation interaction energy computed for the slip planes at z/|b| > 0 is always larger than that for the slip planes at z/|b| < 0, the simultaneous effect of the misfit hardening and the dislocation strengthening in the slip planes at z/|b| > 0 is more obvious than that in the latter. The potential energy in Figure 7d associated with the work generated by the external stress also supports the above suggestion.

**Figure 8:**
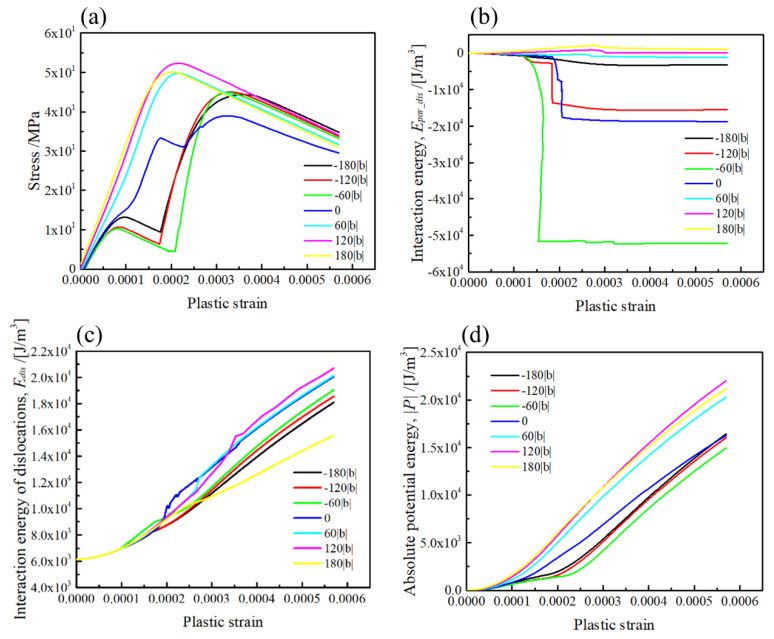
The geometrical effect on the misfit hardening by the [010] rod precipitate. (**a**) Stress–strain curve, (**b**) interaction energy of the precipitate and dislocation, (**c**) interaction energy of the dislocations, (**d**) potential energy due to the dislocation motion. The relative position of the (111) slip plane is ranging from z/|b| = −180 to 180.

The simulation results on the stress–strain curves, and energies for the [010] rod precipitate, are represented in Figure 8 (Type-B variant). As shown in Figure 8a, the stresses necessary for the dislocation glide in the slip planes above the mid-plane (z/|b| > 0) are larger than that below the mid-plane (z/|b| < 0), and the large drops on the stress–strain curves appear in the slip planes at z/|b| < 0, similar to those represented in Figure 8a. According to the interaction energy between the dislocation and precipitate presented in Figure 8b, the interaction energy in the slip planes at z/|b| > 0 is positive and reaches 1.5 × 10^3^ J/m^2^. Meanwhile, the interaction energy is largely decreased in the slip planes at z/|b| < 0, which is attributed to the dislocation motion influenced by the $\sigma_{\mathrm{xz}}$ stress field local distribution in Figure 4. The interaction energy of dislocations in the slip planes at z/|b| > 0 is always larger than that in the slip planes at z/|b| < 0, which implies that the retarding effect by the [010] rod precipitate results in an increase in the dislocation interaction energy for the slip planes at z |b| > 0. As a consequence, the hardening behaviour in the dislocation interacting with the [010] rod precipitate strongly depends on the relative position of the dislocation slip plane and the rod precipitate. The potential energy dependences demonstrated in Figure 8d clearly indicate that the dislocation gliding in the slip planes at z/|b| > 0 requires more work to be generated by the external stress than that for the slip planes at z/|b| > 0.

**Figure 9:**
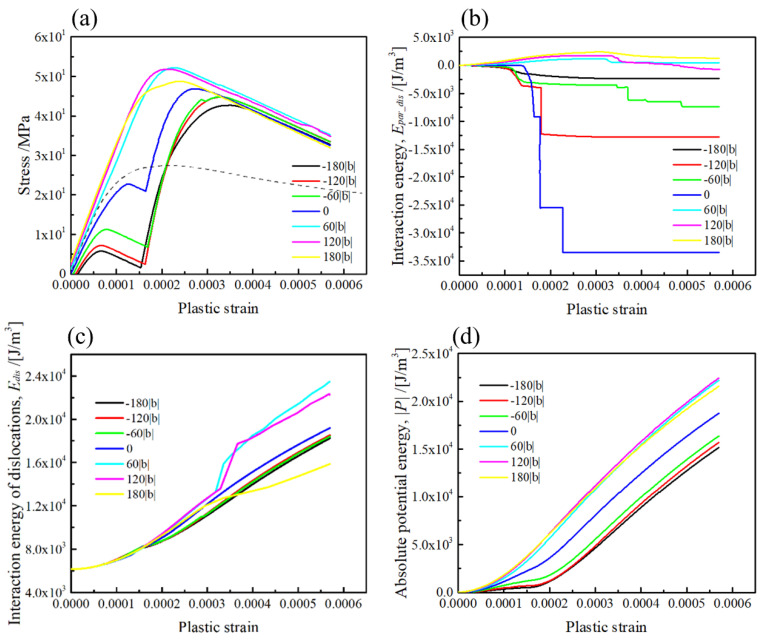
The geometrical effect on the misfit hardening by the [001] rod precipitate. (**a**) Stress–strain curve, (**b**) interaction energy of the precipitate and dislocation, (**c**) interaction energy of the dislocations, (**d**) potential energy due to the dislocation motion. The relative position of the (111) slip plane is ranging from z/|b| = −180 to 180.

The stress–strain curves and energies computed for the [001] rod precipitate are represented in Figure 9 (Type-A variant). As one may see in Figure 9a, the stress necessary for the dislocation glide in the slip planes at z/|b| > 0 is larger than that at z/|b| < 0. Furthermore, the large drop in flow stress can be seen in the stress–strain curves by the slip geometry at z/|b| < 0. Recalling the positive sign of the $\sigma_{\mathrm{xz}}$ in front of the precipitate in the slip planes at z/|b| < 0 (Figure 5), the dislocation motion assisted by the internal stress is responsible for the decrease in the flow stress at the small plastic strain. Moreover, although the magnitude of the $\sigma_{\mathrm{xz}}$ of the rod precipitate in the slip plane at z/|b| = 0 is slightly smaller than that in the other slip planes intersecting with the rod precipitate, the stress necessary for the dislocation glide in the mid-plane (z/|b| = 0) is larger than that below the mid-plane (z/|b| < 0).

According to the interaction energy plots in Figure 9b, the interaction energy with the positive sign reaches 2.0 × 10^3^ J/m^3^ for the slip geometry above the mid-plane (z/|b| > 0), which is an intermediate value between the [100] and [010] variants. Meanwhile, for the slip planes below the mid-plane (z/|b| < 0), negative interaction energy is observed in Figure 9b, which is caused by the positive sign of $\sigma_{\mathrm{xz}}$ in front of the rod precipitate, as shown in Figure 5. The effect of the relative position of the slip plane on the dislocation interaction energy is clearly seen in Figure 9c, where the magnitudes of dislocation interaction energies for z/|b| = 60 and 120 are larger than that obtained in the slip planes below the mid-plane (z/|b| ≤ 0). This is because the positive and negative signs of $\sigma_{\mathrm{xz}}$ in front and behind the rod precipitate (Figure 5) influenced the topological change in the dislocation to reduce the dislocation interaction energy, when the $\sigma_{\mathrm{xz}}$ sign around the precipitate changed with the relative position of the dislocation slip plane.

Regarding the slip planes outside the rod precipitate (z/|b| = −180 or 180), the dislocation suffered a weak retarding force from the rod precipitate, leading to the dislocation motion with the smaller curvature radius. The potential energy associated with the external stress presented in Figure 9d clearly indicates that the potential energy magnitude in the slip planes above the mid-plane (z/|b| ≥ 0) is larger than that in the slip planes below the mid-plane.

## 4. Discussion

As reported in recent simulation works, the effect of misfit strain and stress has a negligible influence on the CRSS associated with the dislocation/precipitate interactions [28,29,30]. However, it is emphasized that the strengthening by <001> rods, as shown in Figure 7, Figure 8 and Figure 9, are apparently geometry-dependent, where the stress necessary for the dislocation overcoming a <001> rod varied with the relative height of the slip plane, z/|b|. This implies that the spatial distribution of three different <001> variants influenced the effective interspacing for dislocation bowing out.

**Figure 10:**
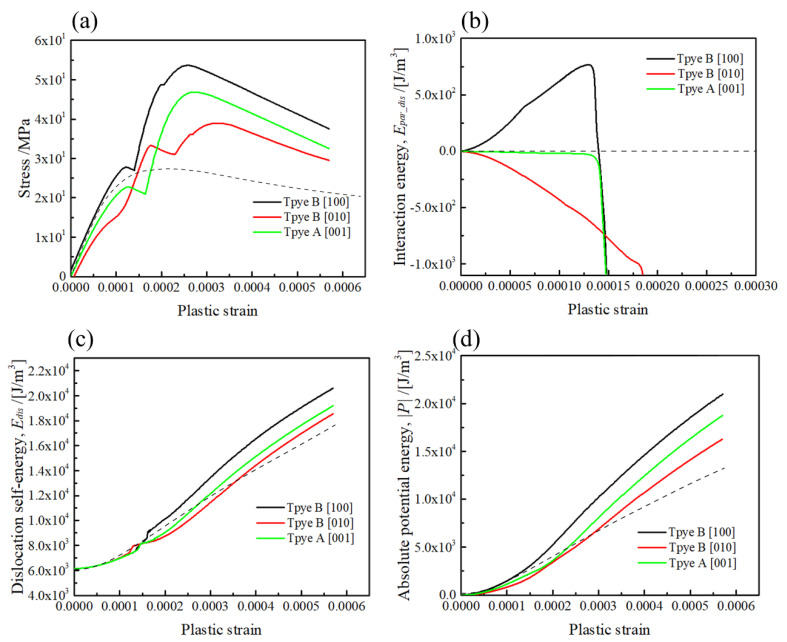
Comparisons of the dislocation interactions computed for the three different {001} rod precipitates at z/|b| = 0. (**a**) Stress–strain curve, (**b**) interaction energy between the precipitate and dislocation, (**c**) interaction energy of the dislocations, (**d**) potential energy due to the dislocation motion. Dotted lines indicate the profiles computed for the dislocation motion without a precipitate.

In order to reveal the orientation-dependent hardening behaviour of the <001> rods, the stress–strain curves and related energy profiles computed for the mid-plane (z/|b| = 0) are replotted in Figure 10. For comparison, the computation results without a <001> rod precipitate are plotted by dotted lines. It can be observed in Figure 10a that the respective stress–strain curves for the [100] and [010] rods (Type-B variants) exhibit stronger and weaker hardening compared to the [001] rod (Type-A variant). The interaction energy profiles in Figure 10b clearly indicate that the hardening rates at the initial part of plastic strain are attributed to the positive and negative interactions caused by different orientations of <001> rods. Moreover, by comparing the stress–strain curve without a rod (dotted line), the dislocation bowing out stress is apparently increased by Type-A and Type-B variants. This indicates that the dislocation bowing out stress can be increased by the misfitting precipitate, unless the local internal stress magnitude in the slip plane is zero, i.e., in the case of the sphere [31] and the {111} plate parallel to the slip plane [32]; the resulting misfit hardening is zero when the dislocation intersects the mid-plane of the precipitate as the Peach–Koehler force associated with the misfitting precipitate is zero. The above facts imply that the bowing out stress of the pinned dislocation segments can be increased by the internal stress of misfitting precipitates as the result of the simultaneous hardening effect by misfitting precipitates and dislocations.

Regarding the average interaction energy (see Appendix A), the interaction between the external stress and the misfit strain of <001> rods and {111} plates vanishes when the geometry of dislocation interaction corresponds to the Type-A variant. However, since the dislocation motion behaviour is locally changed with the internal stress of precipitation variant, as shown in Figure 5 and Figure 6, the Type-A variant shows a medium level of strengthening compared with the Type-B variant.

It is interesting to note that when the dislocation motion direction is changed, the internal stress caused can be alternated by two different orientations of Type-B variants, as shown in Figure 1b,c, i.e., by assuming the opposite sign of dislocation tangent vector (Burger’s vector) in Figure 2, the dislocation motion direction under external stress becomes the opposite. However, since the geometry of positive and negative stresses in front and behind the precipitate is unchanged, the geometry of Type-B variants with strong and weak strengthening can be alternated by assuming the opposite sign of dislocation.

**Figure 11:**
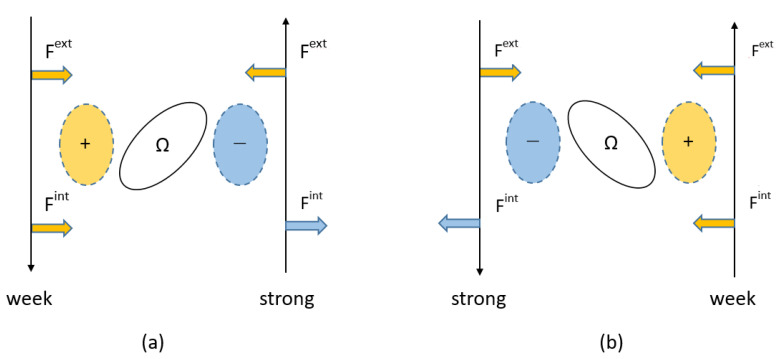
Schematic illustrations of dislocation interaction of Type-B variant with different orientations in (**a**,**b**). Assuming positive external stress in (**a**,**b**), force vectors associated with external and internal stress, *F*^ext^ and *F*^int^, change their directions according to the dislocation tangent vector indicated by upper and lower arrows. Dislocation motion under the positive external stress is always promoted or retarded by positive and negative internal stresses, respectively.

Schematics of the interaction forces with different signs acting on the dislocation are represented in Figure 11. It is important to note that when the sign of the dislocation Burger’s vector changes to the opposite, the force vector associated with the positive external stress becomes opposite as well, as shown in Figure 11a,b. Meanwhile, when the internal stresses with opposite signs exist in front and behind the precipitate, the magnitude of the force vectors associated with the internal stresses can be changed with respect to the motion direction of dislocation, where $F^{\mathrm{ext}}$ and $F^{\mathrm{int}}$ are Peach–Koehler-forces associated with the external and internal stresses, respectively. This fact suggests that the weak and strong orientation relationships in Type-B variants can be alternated by the dislocation motion direction.

Under the ideal condition of the dislocation motion among the oriented precipitates by the single-variant of {001} rods, it is natural to consider that the dislocation motion direction may be chosen in order to interact by the Type-B variant with weak strengthening, as the critical stress to overcome the {001} rod is markedly changed, as shown in Figure 10. This mechanism might be responsible for the strengthening by stress-oriented GP-zones in Al–Cu single crystals, where the Type-B variant of the {001} plates always exhibited low strengthening levels under the compression tests compared with the Type-A variant [19,20]. According to the dislocation dynamics simulation of peak-aged precipitation strengthening by β″ needle-shaped precipitates in Al–Mg–Si alloys, by comparing the simulation models with and without the misfit strain of the precipitate [30], it was concluded that the matrix misfit stress, volume fraction and precipitate shape have small effects on the critical resolved shear. They proposed that the upper strength boundary is approximated by the Orowan looping mechanism. However, since the misfit strain is the only origin of internal stress in the present calculation, the orientation-dependent hardening behaviours observed in the stress–strain responses in Type-A and Type-B variants are attributed to the dislocation motion influenced by the stress field of <001> misfitting precipitates. This fact indicates that the strengthening ability of a certain precipitation variant depends on the types of dislocation interaction when the stress field around the misfitting precipitate is not symmetrical. According to the micromechanics theory, as mentioned in Section 1, although stress disturbance due to the dissimilar stiffness effect, owing to inhomogeneity, is marginal in the case of β″ needles in Al–Mg–Si alloys, the work hardening rate can be influenced by types of {001} variants when the plastic deformation of the matrix phase is dominant to some extent. In such a case, the mismatch of the plastic strain can be similarly treated as the misfit strain, but proportionally changed with the amount of plastic deformation.

Finally, the orientation-dependent hardening behaviours of the <001> rod precipitates are summarised in Table 2. These results are coincident with the average interaction energy, as listed in the Appendix A. This fact implies that the orientation-dependent hardening effect by a single <001> rod precipitate can also be predicted by the macroscopic average of interaction energy.

## 5. Conclusions

In the present work, the stresses caused by the misfitting <001> rod precipitates and the curved dislocation segments were explicitly computed by Green’s function method to investigate the geometry- and orientation-dependent hardening effect by the <001> rod precipitate. The main conclusions are listed as follows:

(1) The stress field of the <001> variants on the dislocation slip plane was greatly changed with the orientation of the rod, which influenced the topological changes of dislocation motion and the resultant stress–strain curves.
(2) Medium strengthening was observed when the Burger’s vector was normal to the longitudinal direction of the [001] rod (Type-A variant), while strong and weak hardening effects were found when Burger’s vector intersected the [100] and [010] rods with the inclined angle of 60 degrees (Type-B variants).
(3) The maximum strengthening and slope of the stress–strain curves for overcoming dislocation in the cases of Type-A and Type-B variants are in good agreement with the prediction of the average interaction energy of the external stress and the misfit strain.

The observed findings of the dislocation interaction of a single <001> rod precipitate suggest that strong and weak orientation relationships always exist under a certain geometry of dislocation slip. In view of the maximum strengthening by multiple precipitates, the strong hardening orientation can be noted as the effective pinning point for the dislocation overcoming the misfitting of <001> rods.

## Figures and Tables

**Table 1:** Parameters of the simulation model.

Parameters	Magnitude
Shear modulus μ	27 GPa
Poisson’s ratio υ	0.3
Young’s modulus E	70 GPa
Magnitude of the Burgers vector |b|	0.286 nm
Burgers vector *b*	[−110]
Slip system	(111) [−110]
Precipitate size	a=119 |b| (34 nm) h=238 |b| (68 nm)
Initial length of dislocation	1400 |b| (400.4 nm)
Constant strain rate	$10^{3}\;s^{-1}$
$\mathrm{Biaxial}\;\mathrm{eigenstrain}\;\varepsilon_{ij}^{*}$	$(\varepsilon_{11}^{*}=\varepsilon_{22}^{*}=0.05,\;\varepsilon_{33}^{*}=0$)

**Table 2:** Orientation dependent hardening of edge dislocation overcoming <001> rod precipitates expressed as the maximum values of shear stresses observed in the dislocation slip planes at z/|b| = 0.

Type of Interaction	Orientation	$\mathrm{Maximum}\;\mathrm{of}\;\mathrm{Flow}\;\mathrm{Stress},\;\sigma_{Max}/\mathrm{MPa}$	Average Interaction Energy
Type-B	[100]	54	>0
Type-B	[010]	39	<0
Type-A	[001]	46	=0

## Data Availability

Not applicable.

## Appendix A

The average interaction energy of the dislocation and the misfitting precipitate can be analysed in terms of the stress acting on the misfitting precipitate (eigenstrain) of the precipitate [32]. Under the dislocation slip system, as defined in Figure 2, basic vectors for the crystal orientations can be expressed by the 3 × 3 matrix, D, as follows:

(A1) $$D=\left[\begin{array}{ccc} \frac{1}{\sqrt{2}} & \frac{1}{\sqrt{6}} & \frac{1}{\sqrt{3}}\\ \frac{-1}{\sqrt{2}} & \frac{1}{\sqrt{6}} & \frac{1}{\sqrt{3}}\\ 0 & \frac{-2}{\sqrt{6}} & \frac{1}{\sqrt{3}} \end{array}\right]$$

In order to achieve the transformed stress–strain of the precipitate in the dislocation coordinate, rotation matrix, $A^{R}$, can be deduced from the multiple of the transpose matrix, $D^{T}$, and coordinate of the <001> precipitate, P, as follows:

(A2) $$A^{R}=D^{T}P\;,$$

where the coordinates for three different orientations of <001> rod precipitates can be simply defined as

(A3) $$P_{\left[100\right]}=\left[\begin{array}{ccc} 0 & 0 & 1\\ 1 & 0 & 0\\ 0 & 1 & 0 \end{array}\right],\;P_{\left[010\right]}=\left[\begin{array}{ccc} 0 & 1 & 0\\ 0 & 0 & 1\\ 1 & 0 & 0 \end{array}\right],\;P_{\left[001\right]}=\left[\begin{array}{ccc} 1 & 0 & 0\\ 0 & 1 & 0\\ 0 & 0 & 1 \end{array}\right]\;.$$

Assuming the biaxial eigenstrains along transverse directions of the <001> rod precipitates, the eigenstrain components in the coordinates of the rod precipitate can be expressed as

(A4) $$\varepsilon_{\mathrm{rod}}^{*}=\varepsilon^{*}\left[\begin{array}{ccc} 0.5 & 0 & 0\\ 0 & 0.5 & 0\\ 0 & 0 & 0 \end{array}\right]\;.$$

By substituting Equation (A3) to Equation (A2), rotation matrix $A^{R}$ for the coordinate translation from the <001> rod precipitates to the (111) dislocation slip plane is obtained. Consequently, the eigenstrains of the <001> rod precipitates in the coordinates of the primary slip system yields

(A5) $$\varepsilon_{\left[100\right]}^{*}=\varepsilon^{*}\left[\begin{array}{ccc} 0.25 & -0.144 & -0.204\\ -0.144 & 0.417 & -0.118\\ -0.204 & -0.117 & 0.333 \end{array}\right],\;\varepsilon_{\left[010\right]}^{*}=\varepsilon^{*}\left[\begin{array}{ccc} 0.25 & 0.144 & 0.204\\ 0.144 & 0.417 & -0.118\\ 0.204 & -0.117 & 0.333 \end{array}\right]\;\mathrm{and}\;\varepsilon_{\left[001\right]}^{*}=\varepsilon^{*}\left[\begin{array}{ccc} 0.5 & 0 & 0\\ 0 & 0.167 & 0.236\\ 0 & 0.236 & 0.333 \end{array}\right]\;.$$

Expressing the average interaction energy associated with the dislocation glide under external shear stress as $\sigma_{\mathrm{xz}}^{\mathrm{ex}}$, strong and weak hardening behaviours are expected by the [100] and [010] rod precipitates, respectively, when the positive misfit strain is $\varepsilon^{*}$:

(A6) $$\begin{array}{c} E_{\left[100\right]}^{int}=-\sigma_{\mathrm{ij}}^{}\varepsilon_{\mathrm{ij}}^{*}=-\sigma_{\mathrm{xz}}^{\mathrm{ex}}\varepsilon_{\mathrm{xz}}^{*}=+0.204\;\sigma_{\mathrm{xz}}^{\mathrm{ex}}\varepsilon^{*}>0\left(\mathrm{Type}-B\;\mathrm{strong}\right),\\ E_{\left[010\right]}^{int}=-\sigma_{\mathrm{ij}}^{}\varepsilon_{\mathrm{ij}}^{*}=-\sigma_{\mathrm{xz}}^{\mathrm{ex}}\varepsilon_{\mathrm{xz}}^{*}=-0.204\;\sigma_{\mathrm{xz}}^{\mathrm{ex}}\varepsilon^{*}<0\;\left(\mathrm{Type}-B\;\mathrm{weak}\right)\;\mathrm{and}\\ E_{\left[001\right]}^{int}=-\sigma_{\mathrm{ij}}^{}\varepsilon_{\mathrm{ij}}^{*}=-\sigma_{\mathrm{xz}}^{\mathrm{ex}}\varepsilon_{\mathrm{xz}}^{*}=0\;\left(\mathrm{Type}-A\right)\;. \end{array}$$

Apparently, the average interaction energy should be zero when the dislocation segment equally interacts with all three <001> variants. In view of local dislocation interaction with the precipitate, effective interspacing needed to overcome the precipitates is influenced by the locations of the <001> variants, because the hardening abilities of Type-A and Type-B variants are different.
